# Examining external control arms in oncology: A scoping review of applications to date

**DOI:** 10.1002/cam4.7447

**Published:** 2024-07-10

**Authors:** Eliya Farah, Matthew Kenney, Matthew T. Warkentin, Winson Y. Cheung, Darren R. Brenner

**Affiliations:** ^1^ Department of Oncology, Cumming School of Medicine University of Calgary Calgary Alberta Canada; ^2^ Department of Community Health Sciences, Cumming School of Medicine University of Calgary Calgary Alberta Canada

**Keywords:** comparative effectiveness, external control arm, oncology, randomized control trial

## Abstract

**Objectives:**

Randomized controlled trials (RCTs) are the gold standard for evaluating the comparative efficacy and safety of new cancer therapies. However, enrolling patients in control arms of clinical trials can be challenging for rare cancers, particularly in the context of precision oncology and targeted therapies. External Control Arms (ECAs) are a potential solution to address these challenges in clinical research design. We conducted a scoping review to explore the use of ECAs in oncology.

**Methods:**

We systematically searched four databases, namely MEDLINE, EMBASE, Web of Science, and Scopus. We screened titles, abstracts, and full texts for eligible articles focusing on patients undergoing therapy for cancer, employing ECAs, and reporting clinical outcomes.

**Results:**

Of the 629 articles screened, 23 were included in this review. The earliest included studies were published in 1996, while most studies were published in the past 5 years. 44% (10/23) of ECAs were employed in blood‐related cancer studies. Geographically, 30% (7/23) of studies were conducted in the United States, 22% (5/23) in Japan, and 9% (2/23) in South Korea. The primary data sources used to construct the ECAs involved pooled data from previous trials (35%, 8/23), administrative health databases (17%, 4/23) and electronic medical records (17%, 4/23). While 52% (12/23) of the studies employed methods to align treatment and ECAs characteristics, 48% (11/23) lacked explicit strategies.

**Conclusion:**

ECAs offer a valuable approach in oncology research, particularly when alternative designs are not feasible. However, careful methodological planning and detailed reporting are essential for meaningful and reliable results.

## 1 INTRODUCTION

Well‐conducted randomized controlled trials (RCTs) remain the gold standard of clinical research; however they often require substantial time for patient recruitment and necessitate a large control group to derive robust conclusions.^1^, ^2^, ^3^ In addition, enrolling patients in a control arm can be challenging, particularly when studying rare diseases with limited patient populations or in the context of precision medicine and targeted therapies, where patiet subgroups with matching baseline characteristics are limited.^4^, ^5^, ^6^ With advancements in precision oncology and the development of targeted therapies, tumor classification based on genetic profiles has led to increased phenotypic stratification.^7^, ^8^, ^9^ This has created additional barriers to patient enrollment for clinical trials, particularly due to limited number of patients within each stratum, which can be further exacerbated in rural areas or small treatment centres.^10^, ^11^, ^12^ As a result, employing active controls (i.e., standard of care) rather than placebos has been recommended when conducting clinical trials.^4^, ^13^ However, the rapid evolution of new cancer therapies often leads to changes in standards of care, potentially disrupting the trial equipoise and affecting the capacity for meaningful comparisons.^4^, ^14^

In navigating these challenges, researchers are increasingly adopting single‐arm trials supplemented with external control arms (ECAs).^15^, ^16^, ^17^ An ECA is an umbrella term encompassing various types of controls that are used when randomization is unfeasible or unethical.^18^ External controls are classified as concurrent controls, enrolled and treated simultaneously with the experimental arm but in a distinct setting, and non‐concurrent controls, collected from retrospective data or studies.^19^, ^20^ Also known as synthetic control arms (SCAs) or historical controls, these arms can act as comparator groups by utilizing external data that have been selected to match the population characteristics in the treatment arm.^18^, ^21^ Data for these controls can be extracted from electronic medical records, publicly available databases, registries, peer‐reviewed literature or other sources.^4^ ECAs can potentially expedite clinical research, reduce costs, and mitigate challenges associated with RCTs.^4^, ^22^, ^23^, ^24^, ^25^ While ECAs offer distinct advantages, their inherent limitations must also be considered. For example, the reliance on historical or external data sources can introduce various biases, including selection bias and confounding.^4^, ^15^, ^20^ In addition, as ECAs' baseline randomization is absent in single‐arm trials, ensuring appropriate design of ECAs is crucial for comparability of patients' baseline risk levels.^15^ Therefore, a comprehensive evaluation of factors like data source, data quality, statistical methods used for matching, and the relevance and reliability of variables measured is necessary to construct methodologically sound ECAs.^4^, ^6^, ^26^, ^27^

Furthermore, the application of health data records or real‐world registries as synthetic controls is becoming increasingly popular in clinical research and has been recognized by various international regulatory bodies, such as the United States Food and Drug Administration (FDA), the Canadian Agency for Drugs and Technologies in Health, as well as the National Institute for Health and Care Excellence.^4^, ^28^ Yet no standard guidelines have been recognized for constructing and/or using synthetic or external control arms. These regulatory bodies, however, have expressed interest in real‐world evidence, suggesting that future guidelines may include more detailed guidance on ECA use. For example, the FDA approved cerliponase alfa treatment for a specific form of Batten disease based on a synthetic control study which compared data from 22 patients in a single‐arm trial with 42 untreated patients.^4^ NICE appraised 22 individual pharmaceutical technologies by employing ECAs to assess comparative clinical efficacy. While the majority (59%) of these ECAs incorporated published RCT data for their external control, 27% relied on observational data.^29^

The rationale for conducting this scoping review arises from the growing utilization of ECAs in oncology research, aiming to address the challenges encountered in traditional RCTs. Unlike individual literature reviews that often focus on specific aspects of ECAs, such as applications, limitations, data sources, methods, and designs, this scoping review aims to provide a comprehensive synthesis that integrates insights and outcomes from various studies utilizing ECAs in their research design. By consolidating and synthesizing the existing literature, we aim to describe current applications and inform future study designs.

## 2 METHODS

We conducted a scoping review instead of a systematic review in compliance with the guidelines presented by Munn et al.^30^ The database was first searched on November 10, 2022, while a manual search was conducted on December 10, 2022. Considering the increasing adoption of ECAs in research design, we aimed to explore the current knowledge and evidentiary base in this specific field.

### 2.1 Search strategy and selection criteria

We systematically searched four electronic databases, including MEDLINE (Ovid), EMBASE, Web of Science, and Scopus, to identify relevant peer‐reviewed studies on this topic. Our search strategy was validated by a librarian (C.M.) at the University of Calgary Health Sciences Library. The strategy consisted of the index keywords (cancer and synthetic control arms), along with their associated MeSH and iterative search terms (Table S1). We did not apply language restrictions to ensure that relevant studies published in other languages were included in the synthesis.

### 2.2 Eligibility assessment

We employed a broad and inclusive approach to our eligibility criteria, with the intention of encompassing a wide array of articles that highlight the applications of ECAs in oncology. To be included in our review, an article must satisfy the following criteria: (1) involved patients diagnosed with cancer of any type, (2) employed a synthetic control arm in the design, and (3) reported clinical outcome(s) in the treatment arm and the synthetic control arm. We excluded literature reviews, gray literature, posters, and commentaries. Studies focusing on the assessment of multiple trials concurrently, rather than evaluating the efficacy of a single intervention from a specific single arm/cohort study, were also excluded. These included master protocol basket trials (i.e., basket and umbrella trials) which we made references to in the discussion section to provide context. Lastly, we excluded articles centered on the methodology of executing ECAs, including those addressing the matching of baseline characteristics.

We imported all records into EndNote X9^31^ reference management software to remove any duplicates. The remaining records were then transferred to the COVIDENCE web platform.^32^ We further filtered out any remaining duplicates and initiated title and abstract screening. In the first round, two reviewers (E.F. and M.K.) independently screened the titles and the abstracts of the included records. Discrepancies, accounting for only 5% (31/629) of all studies, were resolved through consensus. Following primary screening, full‐text eligibility screening was performed and validated independently. Reference lists of records included in the full‐text eligibility screening were manually searched and validated for additional records that met the eligibility criteria.

### 2.3 Data abstraction

Data from all included records were independently abstracted by the same two reviewers. One reviewer then verified all abstracted data and discrepancies were resolved through consensus. From all included articles, we extracted the following variables: authors' names, cancer site and type, stage, source of real‐world data used to construct the ECAs, and study design. We also gathered data on the countries where the studies were conducted. These locations also coincided with the areas from which the patient experimental group was enrolled (Table 1). We also extracted additional variables related to the study design and the statistical methods used. These included specific covariates used for adjustment, the methods employed for such adjustments, primary outcomes in their respective units (i.e., odds ratio, percentage, time, etc.), and their definitions. Moreover, we retrieved pooled estimates with their 95% confidence intervals and their corresponding *p*‐values (Table 2).

**TABLE 1:** Overview of the 23 studies utilizing synesthetic control arms in various cancer types, stages, and data sources.

Cancer site	Cancer type	Cancer stage(s)	Source of real‐world data	Study design	Country	First author	Year of publication																											
Bile Duct	Biliary tract	III–IV	Pooled data of previous trials	Open‐labeled, single‐arm phase II trial	Japan	Matsuyama et al.																			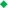									
	FLT3+ Acute myeloid leukemia	I–III	Electronic medical records	Single center, retrospective cohort study	United States	Bazzell et al.																											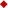	
	B‐precursor Philadelphia chromosome‐negative relapsed/refractory acute lymphoblastic Leukemia	Not reported (NR)	External databases (European national study groups and large individual sites from Europe and the United States)	Single‐arm, phase II trial	United States and Europe	Gökbuget et al. ^a^																					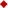							
	Multiple myeloma	II–IV	Pooled data of previous trials	Retrospective cohort study	Czech Republic	Jelinek et al.																							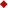					
	Multiple myeloma	I, IIA, IIIA, IIIB	Registry	Prospective single arm trial	United States	Laszlo et al.																									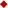			
Blood	Philadelphia chromosome‐positive acute lymphoblastic leukemia	I–III	Administrative health database	Single arm, phase II trial	South Korea	Lee et al.										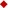																		
	Relapsed or refractory multiple myeloma	I–III	External database (flatiron Health/Foundation Medicine Inc Clinico‐Genomic Database)	Retrospective cohort study	United States	Martin et al.																											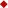	
	Childhood Leukemia	NR	Registry	Randomized controlled trial	Russia	Shelikhova et al.																										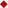		
	Myeloid Leukemia	I–IV	Pooled data of previous trial	Multicenter, open‐label, nonrandomized trial	Germany	Uffmann et al.																						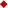						
	Multiple myeloma	I–IV	Electronic medical record	Retrospective cohort study	United States	Usmani et al.																						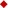						
	Relapsed or refractory multiple myeloma	I–III	Pooled data of previous trials	Retrospective Cohort Study	United States	Weisel et al. ^a^																											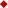	
	Node‐Negative Breast	I–II	Administrative health database & registry	Retrospective comparative cohort study	United States	Cox et al.											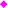																	
Breast	Breast	Clinical (0‐IV), T‐stages (TX, NX, MX)	Administrative health database	Retrospective cohort study	United States	Cupples et al.										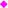																		
	Hormone‐receptor‐positive early breast cancer	I–II	Pooled data from previous trial	Prospective, single‐arm study	United States	Sun et al. ^a^																									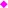			
	Colorectal	IV	Pooled data from previous trial	Non‐randomized, open‐label, interventional study	South Korea	Jo et al.																		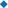										
Colorectal	Colorectal	IV	Electronic medical record	Nonrandomized, multi‐center, open‐label multi‐basket phase IIa trial	Japan	Narita et al.																											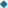	
	Rectal	II–IV	Not stated	Phase‐II clinical trial	Iran	Omidvari et al.																				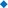								
Gastrointestinal	Gastric with ulcer scars	I–II	Administrative health databas	Single‐center, retrospective, comparative study	Japan	Higuchi et al.																		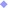										
Lung	Anaplastic lymphoma kinase‐positive non‐small‐cell lung cancer	IIIB, IV	Electronic medical record	Two single‐arm Phase II trials	Multiple countries	Davies et al. ^a^																												
	RET Fusion‐positive non‐small cell lung cancer	I–IV	Pooled data of previous trials	multi‐cohort, open‐label, phase I/II study	Multiple countries	Popat et al. ^a^																									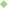	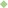		
Renal	Renal cell carcinoma	III–IV	External database (Large individual sites from both Europe and the United States)	Retrospective comparative cohort study	Japan	Onishi et al.	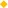																											
Skin	Melanoma	IIIA or IIIB, AJCC: III	Registry	Open labeled, multicentre, randomized phase II trial	Switzerland	Lienard et al.					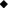																							
Multiple	Gastroenterological, Gynecologic, Head and neck non‐melanoma, Lung, Other rare, Sarcoma, Skin, Unknown, Urologic	IV	Administrative health database	Retrospective cohort study	Australia	O'Haire et al.																										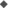		
							1995			2000					2005					2010					2015					2020				

*Note*: Advanced bile duct cancer: Stages III–IV; Advanced multiple cancers: Stage IV; Advanced renal cancer: Stages III–IV; Early‐stage GI cancer: Stages I–II; Metastatic colorectal cancer: Stage IV.

Abbreviation: NR, Not reported.

^a^ Article manually added.

**TABLE 2:** Summary of design and statistical parameters including covariate adjustments, methods, and outcomes for the 23 included synesthetic control arm studies.

Authors	Covariates adjusted	Adjustment method(s)	Primary outcomes (unit)	Definition(s)	Pooled estimate [95% Confidence Interval] (Treatment vs Control)	*p*‐value
Bazzell et al., 2022	Age, complete remission (CR) status after induction, Daunorubicin dose, Karyotype, NPM1 mutation status	Propensity score matching	EFS (months)	Time from start of induction therapy to relapse, failure to attain a CR after induction therapy, or death	Unadjusted: Not reached (NRe) vs. 8.0 [0.0–28.96]	0.102
					Adjusted: NRe vs. 8.0 [1.7–14.3]	0.343
			OS (months)	Time from start of induction therapy to death	Unadjusted: NRe vs. 25.7 [6.8–44.7]	0.128
					Adjusted: NRe vs. 28.7 [0.0–75.9]	0.752
			ORR (%)	Proportion of patients who achieved either a CR or complete remission with incomplete count recovery (CRi)	Unadjusted: 16.7% vs. 34.0%	0.075
					Adjusted: 20.8% vs. 28.0%	1.00
Cox et al., 2006	Not reported (NR)	NR	OS	Not defined	‐	0.98
			DFS	Not defined	‐	0.48
Cupples et al., 2005	NR	NR	Recall rates (%)	The proportion of patients in whom biopsy is generated among the total number of patients screened	8.31 vs. 7.69	0.092
			Biopsy rates (%)	The proportion of patients recalled from the screening test for diagnostic workup among the total number of patients screened	1.46 vs. 1.37	0.613
			Cancer detection rate (%)	The proportion of cancers detected per 1000 patients screened	4.30 vs. 3.60	0.532
			Detection rate of invasive cancers of 1.0 cm or less (%)	The proportion of invasive cancers of 1.0 cm or less detected per 1000 patients screened	1.34 vs. 0.51	0.069
			Detection rate of in situ cancers (%)	The proportion of in situ cancers per 1000 patients screened	1.19 vs. 1.2	0.849
			Positive predictive value for biopsies done (%)	‐	29.20 vs. 26.90	0.708
Davies et al., 2018 ^a^	Age, gender, race, stage at initial diagnosis, prior lines of therapy	Propensity score weighting—Inverse probability of treatment weights (IPTW)	OS (months)	The time from the date of initiation of alectinib or ceritinib until death from any cause	Unadjusted: 26.0 [21.0–NR] vs. 16.0 [12.0–NR]	NR
					Adjusted: 24 [21.0‐NR] vs. 16.0 [16.0–19.0]	
Gökbuget et al., 2016 ^a^	Age, sex, duration between initial diagnosis and salvage therapy, region, prior hematopoietic stem cell transplant, prior number of salvage therapies, primary refractory and in first salvage, refractory to last salvage therapy	Weighted analysis (outcomes from the historical data were weighted according to the frequency of distribution of prognostic baseline factors)	CR after salvage therapy (%)	European sites: bone marrow blasts <5% and no peripheral blast cells or extramedullary manifestations US sites: use CR criteria as published for acute myeloid leukemia, involving the complete recovery of peripheral counts	43% [36–50] vs. 24% [20–27]	NR
			OS (months)	The time from the start of last salvage therapy to death from any cause.	6.10 [4.20–7.50] vs.3.30 [2.80–3.60]	
		Propensity score matching (baseline factors)	Odds Ratio: CR after salvage therapy (%)	European sites: bone marrow blasts <5% and no peripheral blast cells or extramedullary manifestations US sites: use CR criteria as published for acute myeloid leukemia, involving the complete recovery of peripheral counts	2.68 [1.67–4.31]	
			Odds Ratio: OS (months)	The time from the start of last salvage therapy to death from any cause.	0.54 [0.39–0.73]	
Higuchi et al., 2013	NR	NR	En bloc resection rate (%)	The endoscopic resection of an entire lesion in a single procedure	100.0 vs. 89.0	0.06
			Complete resection rate (%)	The endoscopic resection of an entire lesion in a single procedure, with no histopathologic evidence of tumor at the resection margins.	90.0 vs. 78.0	0.21
			Median treatment time (minutes)	The length of time from the start of treatment that half of the patients in a group of patients diagnosed with the disease are still alive	80.0 vs. 101.0	0.22
			Adverse events after ESD (%)	Not defined	30.0 vs. 37.0	NR
			Adverse events during ESD (%)	Not defined	17.0 vs. 19.0	NR
Jelinek et al., 2018	Age, albumin level, β2‐microglobulin, level incidence of thrombocytopenia (defined as platelet count <150/mL), lines of therapy, prior pomalidomide/carfilzomib exposure, sex, refractory status	Multivariate Cox proportional hazards regression modeling	PFS (months)	Time between the date of the first dose of daratumumab and either disease progression or death	Unadjusted HR, 1.14 [0.94–1.39]	NR
					Adjusted HR, 0.61 [0.48–0.78]	NR
			OS (months)	Number of days from treatment initiation to death; patients were censored at the last known date that the patient was alive	Unadjusted HR = 0.79 [0.56–1.12]	0.192
					Adjusted HR = 0.33 [0.21–0.52]	<0.001
Jo et al., 2013	Addition of irinotecan, age, cetuximab administration schedule, gender, primary tumor site, topical vitamin K1 application	Multivariate Cox regression analysis	Incidence of grade ≥2 acneiform rash at 4 weeks in patients treated with cetuximab (%)	Acneiform rash is defined according to the National Cancer Institute Common Terminology Criteria, version 3.0	55.5 vs. 42.5	NR
			Severity of acneiform rash (%)	Percentage of grade ≥2 acneiform rash in patients treated with cetuximab	55.7 vs. 55	NR
			Median time to grade ≥1 acneiform rash in patients treated with cetuximab (weeks)	Time from treatment of cetuximab to reaching grade ≥1 acneiform rash	2.0 vs. 2.0	0.285
			Median time to grade ≥2 acneiform rash in patients treated with cetuximab (weeks)	Time from treatment of cetuximab to reaching grade ≥2 acneiform rash	4.0 vs. 6.0	0.340
Laszlo et al., 2020	NR	NR	Total cost per patient (Euros, €)	Total costs were calculated from preapheresis, periapheresis, and post apheresis costs	12,690 vs. 16,088	0.07
			Average CD34+ Collected Total × 10^6^/kg	Average count of CD34+ cells collected	5.8 vs. 10.6	0.004
Lee et al., 2005	NR	NR	Probability of relapse (%)	The reappearance of more than 5% leukemic cells in bone marrow (BM) aspirates or extramedullary leukemia in patients with previously documented complete remission (CR)	3.8 vs. 45.7	0.803
			Probability of NRM (%)	Death occurring in relapse‐free patients	95.6 vs. 59.3	0.134
			Probability of DFS (%)	Not defined	78.1 vs. 38.7	<0.001
			Probability of OS (%)	Not defined	78.1 vs. 38.7	<0.001
			First complete remission (CR1) after induction therapy (%)	CR was defined as the reconstitution of normal BM cellularity with less than 5% leukemic blasts, together with an absolute neutrophil count of greater than 1.5 109/L and a platelet count greater than 100, 109/L. Molecular CR was defined by a negative pathologic complete response in BM aspirates. CR1 indicates first complete remission	79.3 vs. 81.8	0.803
			Sustained CR1 after consolidation therapy (%)		95.6 vs. 59.3	0.003
			CR1 after salvage therapy (%)		50 vs. 16.7	0.545
			Pretransplantation disease status CR1—Sustained CR1 during consolidation phase plus newly achieved CR1 after the imatinib or salvage chemotherapy (%)		86.2 vs. 51.5	0.004
Lienard et al., 1999	NR	NR	Complete Response, % (TM‐ILP vs. TIM‐ILP vs. M‐ILP)	Not defined	68.8 vs. 78.1 vs. 52.4	NR
			Partial Response, % (TM‐ILP vs. TIM‐ILP vs. M‐ILP)	Not defined	21.9 vs. 21.9 vs. 25.2	NR
			Time to local progression or recurrence, days (TM‐ILP vs. TIM‐ILP vs. M‐ILP)	Not defined	819.0 vs. >705.0 vs. 338.0	NR
Martin et al., 2022	Age, cytogenic profile, international staging, System stage, number of prior lines of therapy (LOTs), refractory status, time to progression on last regimen, years since diagnosis	Propensity score weighting—Inverse probability of treatment weights (IPTW)	PFS	The time from the index date to the date of progression, death, or start of next treatment, whichever occurred first, with the date of last follow‐up used in censoring	Unadjusted HR = 0.20 [0.14–0.28]	<0.0001
					Adjusted HR = 0.18 [0.12–0.27]	<0.0001
			TTNT	The time from the index date to the initiation of the next LOTs or death, whichever occurred first	Unadjusted HR = 0.17 [0.11–0.24]	<0.0001
					Adjusted HR = 0.15 [0.09–0.22]	<0.0001
			OS	The time from the index date to the date of the patient's death. If the patient was still alive or their vital status was unknown, data were censored at the last date known to be alive (CARTITUDE‐1) or the last follow‐up date (maximum of last treatment end date or last visit date) (RW cohort)	Unadjusted HR = 0.28 [0.18–0.45]	<0.0001
					Adjusted HR = 0.25[0.13–0.46]	<0.0001
Matsuyama et al., 2014	NR	NR	Median OS (months)	Time from the day of registration to the day of death from any cause or 1.5 years afterwards	10.1 vs. 7.6	*p* = 0.079 Harrington‐Fleming method: *p* = 0.043
			Median OS by site—Intrahepatic Bile Duct (months)		11.6 vs. 8.7	NR
			Median OS by site—Extrahepatic Bile Duct (months)		18.3 vs. 10.1	NR
			Median OS by site—Gallbladder (months)		8.4 vs. 6.5	NR
			Median OS by site—Vatar papilla (months)		9.8 vs. 9.3	NR
Narita et al., 2022	Age, KRAS Status, number of previous metastatic treatment regimens, previous exposure to anti epidermal growth factor receptor (anti‐EGFR) therapy, time from initial metastatic diagnosis to index date, tumor site	Propensity score weighting—Inverse probability of treatment weights	OS preweighting population	Time from index treatment initiation to death	Unadjusted HR: 0.90 ([0.46–1.77)]	NR
			OS postweighting population		Unadjusted HR: 1.04 [0.43–3.94]	NR
					Adjusted HR: 0.73 [0.18–3.90]	NR
O'Haire et al., 2021	Age, cancer type, Eastern Cooperative Oncology Group failed lines of systemic treatment, remaining reimbursed therapeutic options as per clinician at initial appointment, sex	Propensity score weighting—Inverse probability of treatment weights	OS (months)	Not defined	Unadjusted HR = 0.44 [0.22–0.88]	0.02
					Adjusted HR = 0.92 [0.65–130]	0.63
Omidvari et al., 2015	NR	NR	Pathologic Complete Response (%)	The disappearance of all invasive tumors, either on a macroscopic or a microscopic scale, in the rectum and the lymph nodes	29.4 vs. 11.7	*p* < 0.028
Onishi et al., 1996	NR	NR	Frequency of human leukocyte class II HLA‐DR antigens: 24	Not defined	0.48	0.041
			Frequency of human leukocyte class I HLA‐B antigens‐w60		6.14	0.00453
			Frequency of human leukocyte class I HLA‐C antigens‐w4		3.95	0.015
			Frequency of human leukocyte class I HLA‐B antigens‐35		5.20	0.0032
			Frequency of human leukocyte class I HLA‐B antigens‐w46		4.55	0.0071
			Frequency of human leukocyte class I HLA‐B antigens‐w48		38.59	0.00039
			Frequency of human leukocyte class I HLA‐B antigens‐w60		6.14	0.00453
			Frequency of human leukocyte class II HLA‐DR antigens‐w8		3.07	0.030
			Frequency of human leukocyte class II HLA‐DR antigens‐5		0.09	0.019
Popat et al., 2022 ^a^	Age, sex, smoking at baseline, Eastern Cooperative Oncology Group (ECOG), time from initial diagnosis to first dose, stage at initial diagnosis, race, brain/central nervous system metastasis only	Propensity score weighting—Inverse probability of treatment weights	Hazard ratio: OS (months) − 1 L pembrolizumab + chemotherapy	Not defined	0.36 [0.21–0.64]	NR
			Hazard ratio: PFS (months) − 1 L pembrolizumab + chemotherapy		0.50 [0.36–0.70]	
			Hazard ratio: Time to treatment discontinuation (months) − 1 L pembrolizumab + chemotherapy		0.50 [0.36–0.70]	
			Hazard ratio: OS (months) − 1 L pembrolizumab		0.33 [0.18–0.61]	
			Hazard ratio: PFS (months) − 1 L pembrolizumab		0.49 [0.33–0.73]	
			Hazard ratio: Time to treatment discontinuation (months) − 1 L pembrolizumab		0.47 [0.31–0.70]	
Sun et al., 2020 ^a^	Age, BMI, nodal status, prior AI received, prior chemotherapy received	Propensity score matching	Method 1: 3‐year breast cancer free interval	The time from enrollment in the study to the first invasive breast cancer event (local, regional, or distant recurrence or a new invasive contralateral breast cancer)	5.8 vs. 9.50 [7.90–11.10]	NR
			Method 2: 3‐year breast cancer free interval		5.8 vs. 9.40 [7.80–10.90]	
Shelikhova et al., 2021	NR	NR	2‐year nonrelapse mortality (%)	Not defined	2.0 vs. 13.0	0.002
			2‐year incidence of relapse (%)	Not defined	26.0 vs. 19.0	0.20
			2‐year OS (%)	Time from hematopoietic stem cell transplantation (HSCT) to death from any cause	81.0 vs. 76.0	0.32
			2‐year EFS (%)	Relapse, death from any cause and graft rejection were considered events of interest. Calculated from date of HSCT until either relapse, death, or date of last follow‐up alive and in complete remission	71.0 vs. 67.0	0.39
			Grade II‐IV graft‐versus‐host disease (%)	Not defined	13.0 vs. 16.0	0.23
			Grade III‐IV graft‐versus‐host disease (%)	Not defined	6.0 vs. 4.0	0.40
			Chronic graft‐versus‐host disease (%)	Not defined	7.0 vs. 13.0	0.07
			Incidence of Cytomegalovirus viremia (%)	Not defined	51.0 vs. 54.0	0.50
Uffmann et al., 2017	NR	NR	5‐year EFS (%)	The time from diagnosis to the first event or last follow‐up. Events were death from any cause, failure to achieve remission, relapse, and secondary malignancy. Failure to achieve remission was considered as an event on day 0	87.0 vs. 89.0	0.71
			5‐year OS (%)	The time of diagnosis to death from any cause or last follow‐up.	89.0 vs. 90.0	0.64
			Cumulative incidence of relapse/nonresponse (CIR/NR) (%)	Calculated by the method of Kalbfleisch and Prentice and compared with the Gray test	6.0 vs. 6.0	0.03
			Severe adverse events	Common Terminology Criteria for Adverse Events v3.0 grade III or higher	RR = 0.56 [0.38–0.82]	NR
			Therapy‐related mortality (%)	Not defined	2.9 vs. 5.0	0.276
Usmani et al., 2017	Age, albumin level, exposure to prior therapies, gender, hemoglobin level, lines of therapy (LOTs), refractory status	Multivariate proportional hazards regression modeling	OS	For patients identified in the IMS LifeLink or OPTUM databases, OS from the start of the last LOTs was defined based on death or loss to follow‐up more than 30 days prior to the study end date. For patients in the GEN501 and SIRIUS studies, OS was defined as the number of days from the first dose of daratumumab to death	Unadjusted HR = 0.46 [0.35–0.59]	<0.001
					Adjusted HR = 0.33 [0.24–0.46]	<0.001
Weisel et al., 2021 ^a^	Refractory status, International Staging System (ISS) stage, cytogenetic profile, time to progression on last regimen, extramedullary plasmacytoma, number of prior lines of therapy (LOTs), years since multiple myeloma diagnosis, age, hemoglobin, lactate dehydrogenase levels, prior stem cell transplant, Eastern Cooperative Oncology Group (ECOG) status, race, sex, type of multiple myeloma (MM)	Inverse probability of treatment weighting (IPTW)	PFS	The duration from the index date to the date of progression or death, whichever occurred first	Unadjusted HR = 0.28 [0.20–0.41]	<0.0001
					Adjusted HR = 0.24 [0.15–0.37]	<0.0001
			TTNT	The time from the index date to the initiation of the next LOTs or death, whichever occurred first, with the last known date alive used in censoring	Unadjusted HR = 0.17 [0.09–0.22]	<0.0001
					Adjusted HR = 0.14 [0.09–0.22]	<0.0001
			OS	The duration from the index date to the date of death. Patients who remained alive at the data cut‐off or had an unknown vital status were censored at the last known date alive	Unadjusted HR = 0.29 [0.19–0.47]	<0.0001
					Adjusted HR = 0.21 [0.13–0.35]	<0.0001

Abbreviations: Anti‐EGFR, anti‐epidermal growth factor receptor; BM, bone marrow; CIR/NR, cumulative incidence of relapse/nonresponse; CR, complete remission; CR1, first complete remission; CRi, complete remission with incomplete count recovery; DFS, disease‐free survival; EFS, event‐free survival; ESD, endoscopic submucosal dissection; HLA, human leukocyte antigens; HR, hazard ratio; LDRBT, low‐dose‐rate endorectal brachytherapy; LOTs, lines of therapy; M‐ILP, melphalan; NR, not reported; NRe, not reached; NRM, nonrelapse mortality; OS, overall survival; ORR, overall response rate; PFS, progression‐free survival; TM‐ILP, drug regimen consisting of TNFα and melphalan; TIM‐ILP, three‐drug regimen consisting of TFNα, melphalan, and INFy.

^a^ Article manually added.

We extracted more granular information in the supplementary materials including sample size, aim of each study and the specific inclusion and exclusion criteria that were applied. We also recorded the date of data collection, the type of intervention administered, and the line of therapy (first line, second line, etc.) (Table S2).

## 3 RESULTS

### 3.1 Studies Identified

**FIGURE 1:**
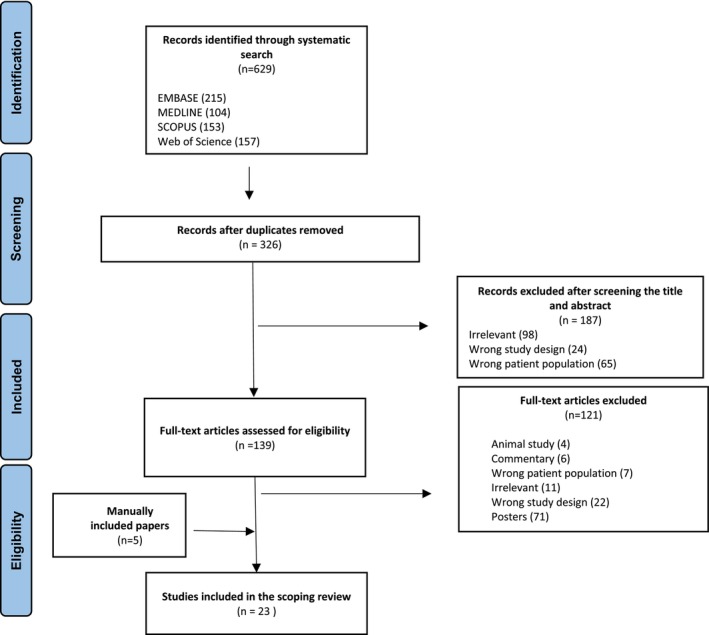
Preferred Reporting Items for Systematic Reviews and Meta‐Analyses (PRISMA) flow diagram for the literature search and study selection.

Figure 1 presents the search results for relevant literature and the screening process. Of the 629 records identified, 303 duplicates were removed. Based on title and abstract, 188 were excluded, leaving 139 full‐text articles to be retrieved and assessed for eligibility. Of these, 18 were included and 121 were excluded for reasons outlined in Figure 1. An additional 5 papers were manually included, for a total of 23 articles in this scoping review.

### 3.2 Timeline

In this compilation of studies, we observed an increasing application of ECAs for oncology research in recent years. The earliest study reported was by Onishi et al.^33^ in 1996, while the most recent studies took place in 2022^26^, ^34^, ^35^, ^36^ (Table 1). During the 1990s, a total of 9% (2/23) of included studies were published.^33^, ^37^ In the subsequent decade, the 2000s, a moderate increase in the application of ECAs was observed, with a total of three studies published, accounting for 13% (3/23).^38^, ^39^, ^40^ The most notable growth in publications occurred in the 2010s, as demonstrated by the 10 studies published during that period, accounting for 43% (10/23).^41^, ^42^, ^43^, ^44^, ^45^, ^46^, ^47^, ^48^, ^49^ This trend appears to continue into the 2020s, with 35% (8/23) studies already completed in the first few years of the decade^26^, ^28^, ^34^, ^35^, ^36^, ^50^, ^51^, ^52^, ^53^ (Table 1).

### 3.3 Disease site and stage

Among the 23 included studies (Table 1), 44% (10/23) investigated blood‐related cancers,^34^, ^35^, ^39^, ^43^, ^45^, ^48^, ^49^, ^50^, ^52^, ^53^ 13% (3/23) of the studies addressed colorectal,^36^, ^42^, ^46^ and breast cancer,^38^, ^40^, ^51^ respectively, while 9% (2/23) examined lung cancer.^26^, ^44^ Bile duct and biliary tract,^41^ gastrointestinal,^47^ renal,^33^ and skin cancers^37^ each constituted 4% (1/23) of the studies, while a single study 4% (1/23) encompassed multiple cancer types.^28^ We observed that the application of ECAs is predominantly focused on rare cancers (i.e., blood cancers) with malignancies defined by unique molecular types or mutations.

Furthermore, ECAs were applied across various cancer stages, from early to advanced. Notably, 39% (9/23) studies specifically addressed advanced stages III–IV.^26^, ^33^, ^36^, ^39^, ^41^, ^44^, ^46^, ^48^, ^51^, ^52^ Meanwhile, 22% (5/23) included a broader range of stages, spanning from early (I) to advanced (III).^35^, ^43^, ^45^, ^50^, ^53^ In a more focused context, 13% (3/23) of the studies focused on early stages I–II,^36^, ^51^ while an additional 13% (3/23) included stages 0–IV.^28^, ^38^ Finally, 9% (2/23) studies did not specify the stages of the cancers investigated^34^, ^37^ and 4% (1/23) reported stages II‐IV.^44^

### 3.4 Country of study conduct

The majority of the 23 studies were conducted in the United States and Japan, coinciding with the geographical location of the experimental group patients included in the studies. 30% (7/23) of the studies originated in the United States,^34^, ^35^, ^38^, ^40^, ^49^, ^50^, ^51^ while 22% (5/23) were conducted in Japan,^33^, ^36^, ^41^, ^42^, ^47^ and 9% (2/23) in South Korea.^39^, ^46^ The remaining 39% (9/23) of the studies were conducted in the Czech Republic, Iran, Russia, Germany, Switzerland, Australia among other countries^26^, ^28^, ^37^, ^42^, ^44^, ^48^, ^52^ (Table 1).

### 3.5 Data types

Pooled data from past trials were the primary data sources used in constructing the ECAs, featured in 30% (7/23) of the studies.^26^, ^41^, ^45^, ^46^, ^48^, ^51^, ^53^ Administrative health databases^28^, ^38^, ^39^, ^47^ and electronic medical records^34^, ^36^, ^44^, ^49^ each contributed to 17% (4/23) of the studies. An additional 13% (3/23) of controls were derived from registries^37^, ^50^, ^52^, and 4% (1/23) did not specify the data source.^42^ Moreover, 13% (3/23) drew from external databases,^33^, ^35^, ^43^ while an equal percentage 4% (1/23) collected from both administrative health databases and registries^40^ (Table 1).

### 3.6 Matching characteristics

Our analysis revealed that 13% (3/23) of the studies employed propensity score matching as an adjustment method to match the characteristics of the treatment arm with those of the ECAs.^34^, ^43^, ^51^ Meanwhile, 26% (6/23) utilized propensity score weighting—inverse probability of treatment weighting.^26^, ^28^, ^35^, ^36^, ^44^, ^53^ In addition, 13% (3/23) used multivariate Cox proportional hazards regression modeling for adjustments.^45^, ^46^, ^49^ On the contrary, 48% (11/23) of the studies did not provide explicit details about covariate adjustments or any specific strategy to ensure congruence between the ECAs and the treatment arm.^33^, ^37^, ^38^, ^39^, ^40^, ^41^, ^42^, ^47^, ^48^, ^50^, ^52^ Table S2 offers further comprehensive insights into these studies.

### 3.7 Data collection timeline

We observed that the majority of studies, with a few exceptions, maintained a close temporal alignment between the data collection periods for the ECAs and the treatment arm. In the study by Jo et al., a gap of approximately 2 years separated the data collection for the treatment arm (October 2010–January 2012) and the ECAs (March 2008–October 2009).^46^ Similarly, Higuchi et al. encountered a comparable gap, where the treatment arm data were collected from October 2008 to May 2012, while the ECAs data spanned from January 2005 to October 2008.^47^ A more substantial temporal gap was evident in the study conducted by O'Haire et al., with nearly a 7‐year period between the treatment arm (May 2015–August 2017) and the ECAs data (November 2008–November 2015).^28^

### 3.8 Line of therapy

With respect to the line of therapy used in the trial intervention, 50% (9/18) evaluated first‐line (1 L) therapy.^26^, ^37^, ^39^, ^40^, ^41^, ^42^, ^46^, ^50^, ^52^ Furthermore, 11% (2/18) of studies examined second‐line (≥2 L) therapy subsequent treatment stages.^34^, ^49^ An additional 39% (7/18) of the studies involved interventions that spanned multiple lines of therapy, reflecting a range of treatment strategies.^35^, ^36^, ^43^, ^44^, ^45^, ^48^ Moreover, 9% (2/23) of the studies^33^, ^51^ did not explicitly report the lines of therapy (Table S2). Whereas 13% (3/23) of the studies focused on screening procedures.^28^, ^38^, ^47^

## 4 DISCUSSION

The adoption of ECAs has been driven by the challenges faced by traditional RCTs, particularly in studies involving rare cancers or precision medicine. While ECAs offer solutions to certain limitations in clinical research, they also present methodological challenges. Although these challenges are not comprehensively discussed in this paper, they generally stem from concerns related to data source reliability, potential biases, and the comparability of controls. The aim of this scoping review was to investigate the breadth of research conducted in the scope of ECAs within oncology and to provide a thorough summary of the peer‐reviewed studies in this area. After rigorous screening, 23 papers were included, all of which specifically integrated ECAs as comparator groups for a single intervention arm.

Our analysis underscores a notable increase in the adoption of ECAs in oncology research, with 65% (18/23) of our articles published since the 2010s. These findings align with similar studies indicating a surge in publications over past two decades.^17^, ^19^, ^54^ This rise may reflect a broader acceptance of ECAs by both pharmaceutical companies and health technology assessment agencies.^4^, ^18^, ^19^, ^55^, ^56^ A notable number of these studies, 44% (10/23), were centered on rare blood‐related cancers identified by distinctive genetic markers, such as FLT3+ in acute myeloid leukemia and the Philadelphia chromosome in acute lymphoblastic leukemia.^34^, ^35^, ^39^, ^43^, ^45^, ^48^, ^49^, ^50^, ^52^, ^53^ In addition, lung cancers, including the ALK‐positive non‐small‐cell and RET Fusion‐positive variations, constituted about 9% (2/23) of the studies.^26^, ^44^ This upward shift appears to align with the growing emphasis on biomarker testing and targeted therapies.^4^, ^57^ With tumors now frequently categorized based on genetic profiles, leading to more refined phenotypic categorization, conventional trials are facing recruitment challenges.^58^, ^59^ Further, approximately 43% (10/23) of the studies focused on advanced cancer stages (III‐IV). This could be attributed to the unique characteristics of advanced‐stage patients, who may not always be optimal candidates for traditional RCTs due to limited patient pools, specific therapy needs, among other factors. Moreover, approximately 30% (7/23) of the studies were conducted in the United States which is consistent with the country's abundant resources, robust research infrastructure, and extensive initiatives in trial designs.^60^

Based on our findings, the majority of studies (30%, 7/23) utilized pooled data from past trials as primary data sources for constructing ECAs. This observation is consistent with other studies that emphasized the use of external control data from recent RCTs, since they uphold a high standard of data collection and are preferred for constructing reliable ECAs.^4^, ^61^ Contrarily, 17% (4/23) of the studies relied on administrative health databases, while another 17% (4/23) utilized electronic medical records as their data sources. These data types, particularly non‐clinical trial data, although they reflect real‐world clinical practice, they might not guarantee the controlled environment typical of RCTs, potentially introducing heterogeneity and bias.^4^, ^24^, ^62^ With respect to matching control data to the intervention group, 26% (6/23) of the studies applied propensity score weighting for adjustments, indicating a focus on achieving balanced and comparable patient cohorts. However, nearly 48% (11/23) of studies did not provide clear information on covariate adjustments—this concern has been underscored in the existing literature as it relates to the failure in accounting for baselines imbalances.^4^, ^15^, ^17^, ^63^, ^64^ As for the data collection timeline, most studies aimed for a close temporal alignment between the ECAs and the treatment arm. However, temporal gaps in some instances indicate potential challenges, possibly related to data accessibility.

Additional instances of ECAs used to provide contextualization for single‐arm trials have been retrospectively demonstrated in the reanalyses of clinical trials across various cancer sites, including leukemia, Merkel cell carcinoma, myeloma, non‐small cell lung cancer, and early‐stage hormone receptor‐positive breast cancer.^21^, ^61^, ^65^, ^66^ Similarly, the incorporation of ECAs derived from real‐world data to supplement single‐arm oncology trials for regulatory approval and reimbursement decisions has gained popularity.^67^ Various research groups have examined methodological challenges related to ECA design through regulatory case studies, proposing strategies to address these challenges.^65^, ^68^ However, the absence of comprehensive guidance for optimal ECA development from regulatory bodies and health technology assessment (HTA) agencies has led to ambiguity in ECA design principles and uncertainty regarding their consistent acceptance by regulators and HTAs. Establishing reporting guidelines for the use of ECAs should be a priority, particularly as master protocol trials become more prevalent, and the number of single‐arm studies continues to rise. Such guidelines could facilitate standardization and promote consistency in the implementation of ECAs.

While our scoping review, is comprehensive, it is not without limitations. which should be acknowledged. First, the scope of our search strategy might have inadvertently excluded relevant studies due to the specific terms and databases used. Although we aimed for inclusivity, it is possible that some pertinent research was missed, potentially affecting the comprehensiveness of our findings. Additionally, the exclusion of gray literature, posters, and commentaries might have omitted valuable insights or methodological details not covered by peer‐reviewed articles. In our review, we highlighted the use of ECAs within oncology research, acknowledging the growing application of this study design. After our search cut‐off date of November 10, 2022, additional pertinent studies have been published. These include a study on metastatic colorectal cancer and another on ovarian cancer, both of which evaluated the efficacy of immunotherapy in a non‐randomized setting using ECAs.^69^, ^70^ These studies exemplify the continued evolution in the application of ECAs and highlight the potential in contexts where RCTs may not be feasible. The publication of these studies post our search date should be acknowledged as a limitation in our review. Their inclusion in future reviews could provide a more comprehensive understanding of the ECA approach in evaluating the efficacy of emerging therapies like immunotherapy, especially in advanced cancer stages where traditional RCTs face challenges in recruitment. Furthermore, we did not to assess the quality of the included articles, as our primary objective was to consolidate and synthesize studies that specifically focused on the application of ECAs in oncology research. Despite these limitations, our scoping review provides a valuable overview of the current landscape of ECAs in oncology research.

## 5 CONCLUSION

This scoping review examines the current applications of ECAs in oncology research, reflecting on the methodological diversity and the evolving landscape of clinical trials. Our findings reveal a significant geographical spread in ECA usage, predominantly in studies from the United States and Japan. Notably, the primary data sources for ECAs included pooled data from past trials, administrative health databases, and electronic medical records. This diversity in data sourcing underscores the flexibility of ECAs but also introduces potential variabilities that can affect the quality and comparability of the results. While ECAs offer an invaluable resource in situations where traditional RCTs are not feasible, our review also underscores the methodological heterogeneity and the challenges it poses for comparative effectiveness research.

Based on the identified literature base, it is evident that there is a critical need for establishing robust guidelines that address the selection of appropriate data sources, the application of statistical methods for matching, and the overall design and execution of ECAs. Furthermore, guidelines for the consistent reporting of ECAs within the literature are lacking and needed. Such guidelines would not only improve the uptake of ECAs but also enhance their utility in regulatory and clinical decision‐making processes.

## PRÉCIS

The manuscript underscores the growing use of External Control Arms in oncology research for rare cancers and precision medicine, advocating for standardized guidelines to ensure their robust and reliable application. It also stresses the importance of methodological rigor in matching treatment and control groups to enhance the credibility of study outcomes.

## AUTHOR CONTRIBUTIONS

**Eliya Farah:** Conceptualization (lead); data curation (lead); formal analysis (lead); methodology (equal); writing – original draft (lead); writing – review and editing (equal). **Matthew Kenney:** Conceptualization (equal); data curation (equal); formal analysis (equal); methodology (equal); resources (equal); visualization (equal); writing – review and editing (equal). **Matthew T. Warkentin:** Methodology (equal); validation (equal); writing – review and editing (equal). **Winson Y. Cheung:** Conceptualization (equal); methodology (equal); project administration (equal); supervision (lead); validation (equal); writing – review and editing (equal). **Darren R. Brenner:** Conceptualization (equal); formal analysis (supporting); methodology (lead); project administration (lead); resources (equal); supervision (equal); validation (equal); writing – review and editing (equal).

## FUNDING INFORMATION

None reported.

## CONFLICT OF INTEREST STATEMENT

No conflict of interest to disclosed.

## Supporting information


Data S1.

## Data Availability

Data sharing is not applicable to this article as no new data were created or analyzed in this study.
