# Supplementary material for: Examining external control arms in oncology: A scoping review of applications to date
Source: Cancer Med. 2024 Jul 10;13(13):e7447. doi: 10.1002/cam4.7447 (PMC11234289; doi:10.1002/cam4.7447)
Supplement: Supplementary file 1 — Data S1. [file CAM4-13-e7447-s001.docx]

**Supplementary Table 1.** Search strategy used to identify relevant studies on the applications of synthetic control arms in oncology.

The search was performed on 10 November 2022, with no restriction on publication language.

| **Search term** | **Search number** | **Keywords/MeSH terms** | **Statement** | **Databases** |
| --- | --- | --- | --- | --- |
| Cancer | 1 | 'neoplasm'/exp OR (Neoplasms) OR (Cancer) OR (Cancers) OR (Neoplasia) OR (Neoplasm) OR (Tumors) OR (Tumor) OR (Malignancy) OR (Malignancies) OR (Malignant Neoplasms) OR (Malignant Neoplasm) OR (Neoplasm Benign) | [mp=ti, ab, hw, tn, ot, dm, mf, dv, kf, fx, dq, bt, nm, ox, px, rx, an, ui, sy] | EMBASE, MEDLINE, SCOPUS, Web of Science |
| Synthetic Control Arm | 2 | (Historical Control Arm) OR (External Control Arm) OR (Synthetic control arm) OR (Historical control data) OR OR (Companion dataset) | [mp=ti, ab, hw, tn, ot, dm, mf, dv, kf, fx, dq, bt, nm, ox, px, rx, an, ui, sy] | EMBASE, MEDLINE, SCOPUS, Web of Science |
| Aggregation of search terms | 3 | 1 AND 2 | - | - |

**Supplementary Table 2.** Summary of the characteristics and general parameters of the 23 studies utilizing synesthetic control arms in Cancer Research

| **Authors** | **Patient (No.)**  **Total; treatment arm; historical control arm** | **Aim of study** | **Inclusion**  **criteria** | **Exclusion**  **criteria** | **Date of data collected** | | **Intervention** | | **Line of therapy (1L,2L,etc.)** |
| --- | --- | --- | --- | --- | --- | --- | --- | --- | --- |
|  |  |  |  |  | **Treatment**  **arm** | **Historical control arm** | **Treatment**  **arm** | **Historical**  **control arm** |  |
| [Bazzell et al., 2022](https://pubmed.ncbi.nlm.nih.gov/34074182/) | 83, 36, 47 | Determine the effectiveness of deferring the initiation of Midostaurin until after induction therapy and to subsequently utilize sorafenib as maintenance therapy in FLT3+ AML patients compared to historical control patients who did not receive FLT3 inhibitors. | 1. Newly ≥ 18 years of age diagnosed AML patients with FLT3 internal tandem duplications (ITD) / tyrosine kinase domain (TKD) mutations 2. Positive for a FLT3 mutation on molecular analysis 3. Treated with intensive '3+7' daunorubicin and cytarabine induction chemotherapy | 1. Had wild-type FLT3 AML 2. Received induction chemotherapy at an outside institution. 3. Pursued treatment on a clinical trial 4. Had insufficient records to facilitate data collection | January 2010- December 2019 | January 2010- May 2017 | FLT3 inhibitor | SOC | 2L |
| [Cox et al., 2006](https://pubmed.ncbi.nlm.nih.gov/16538416/) | 2447, 1854, 593 | To compare the survival outcomes of node-negative patients who received a sentinel lymph node (SLN) biopsy with historical control data of node-negative patients who received routine complete axillary lymph node dissection (CALND) in the pre-SLN biopsy era. | 1. Patients between the age of 25-94 2. Treated at their institution from January 1986 to 2004 3. Treated with CALND or SLN | 1. Patients with   inflammatory T3 and T4 disease | January 1986-May 2004 | January 1986-May 2004 | SLN Biopsy | CALND + (Lumpectomy or mastectomy) | 1L |
| [Cupples et al., 2005](https://pubmed.ncbi.nlm.nih.gov/16177413/) | 27274, 19402, 7872 | The aim of this study is to assess the effectiveness of computer-aided detection (CAD) system compared to radiologists’ judgment on screening outcomes in a regional mammography program. | 1. Aged 35-90 (40-90 pre CAD, 35-90 CAD) 2. Physician-referred asymptomatic women 3. Patients from the main breast center and three affiliated off-site screening facilities. | clinical symptoms (i.e., palpable lump, skin thickening, nipple discharge) | January 1999-December 2001 | January 1998-December 2001 | Screening mammogram + CAD | Screening mammogram without CAD | NA (screening procedure) |
| *[Davies et al.](https://becarispublishing.com/doi/epdf/10.2217/cer-2018-0032) 2018 | 250, 183, 67 | To compare the overall survival of anaplastic lymphoma kinase-positive non-small-cell lung cancer patients who received alectinib with those who received ceritinib | 1.≥18 years or older with locally advanced or metastatic [ALK](https://www.sciencedirect.com/topics/medicine-and-dentistry/anaplastic-lymphoma-kinase)-positive NSCLC, as assessed by an FDA-  approved [fluorescence in situ hybridization](https://www.sciencedirect.com/topics/medicine-and-dentistry/fluorescence-in-situ-hybridization) test.  2.Eastern Cooperative [Oncology](https://www.sciencedirect.com/topics/medicine-and-dentistry/oncology) Group (ECOG) < 2 or  3.progressed while taking [crizotinib](https://www.sciencedirect.com/topics/medicine-and-dentistry/crizotinib). Patients with asymptomatic baseline CNS metastasis  4. Treated or untreated with radiation and those who had received prior chemotherapy. | 1.Received prior [ALK inhibitor](https://www.sciencedirect.com/topics/medicine-and-dentistry/alk-inhibitor) [treatment](https://www.sciencedirect.com/topics/medicine-and-dentistry/therapeutic-procedure) other than crizotinib | Trial NP28761- January 2016 - October 2014  Trial NP28673-February 2016-August 2014) | January 2011 - December 2014 | Alectinib | Ceritinib | Varies (<4L->4L) |
| *[Gökbuget et al., 2016](https://www.ncbi.nlm.nih.gov/pmc/articles/PMC5056974/pdf/bcj201684a.pdf) | 1328,189, 1139** | Evaluate the results of the single-arm clinical trial for the approval of blinatumomab in the USA and Europe, using a historical comparator study to compare complete remission and overall survival rates with standard salvage chemotherapy in adults with B-precursor, Philadelphia chromosome-negative / refractory acute lymphoblastic leukemia | 1.Adult patients with R/R Ph-negative B-precursor ALL  2. Age ⩾15 years at time of initial diagnosis of ALL, age ⩾18 years at relapse  3. Initial diagnosis of ALL in the year 1990 or later  4. No central nervous system involvement at relapse  5.No isolated extramedullary relapse  6. No previous treatment with blinatumomab.   1. 7. Relapsed within 12 months from initial diagnosis, or relapsed after alloHSCT, or refractory to initial or subsequent treatments, or in second or later relapse. | 1. Patients with a first remission duration of 412 months and remaining in first salvage without further relapse  2.patients for whom only palliative care was recorded or had no verifiable record of salvage therapy | January 2012- October 2013 | January 1990-October 2013 | Blinatumomab | standard of care salvage chemotherapy | Varies (<4L->4L) |
| [Higuchi et al., 2013](https://pubmed.ncbi.nlm.nih.gov/23472995/) | 57,30,27 | Evaluate the efficacy and safety of double-endoscope endoscopic submucosal dissection (ESD) by using a single light source compared to conventional ESD in patients with early gastric cancer accompanied by an ulcer scar. | 1. An endoscopic and histopathologic diagnosis of early gastric tubular or papillary adenocarcinoma with an ulcer scar   An endoscopic diagnosis of intramucosal invasion and a tumor diameter of ≥3 cm on the pre-treatment examination   1. No evidence of distant metastasis or lymph-node metastasis on pre-treatment computed tomography (CT) scanning 2. Double-endoscope ESD was performed up to the end of May 2012 at Kitasato University East Hospital, Sagamihara, Japan | Not reported (NR) | October 2008- May 2012 | January 2005-October 2008 | Double endoscope ESD | Conventional ESD | NA (screening procedure) |
| [Jelinek et al., 2018](https://pubmed.ncbi.nlm.nih.gov/29172760/) | 1120,148,972 | Compare progression-free survival and overall survival of MM patients treated with daratumumab monotherapy or physician's treatment choice using patient-level data of a real-world historical control cohort. | 1. ≥ 18 years old 2. Eastern cooperative Oncology Group performance status ≤ 2 3. Heavily pretreated and/or refractory MM 4. Patients relapsed from or refractory to ≥ 2 prior lines of therapy that included a proteasome inhibitor (PI) and immunomodulatory drugs (IMiD) (GEN501) 5. Patients relapsed from or refractory to ≥3 prior lines of therapy that included a PI and an IMiD (SIRIUS) | NR | March 2008- January 2015 (GEN501)  &  September 2013-January 2015 (SIRIUS) | March 2006-March 2015 | Daratumumab Monotherapy | SOC | Varies (>3L) |
| [Jo et al., 2013](https://pubmed.ncbi.nlm.nih.gov/23238388/) | 101,61,40 | Investigate the efficacy and the safety of vitamin K1 cream for acneiform rash associated with cetuximab using a historical control arm. | 1. Metastatic colorectal cancer (mCRC)   patients scheduled to receive cetuximab-containing chemotherapy   1. Histologically confirmed adenocarcinoma of the colon or rectum 2. Tumors harboring wild type KRAS 3. No prior exposure to an anti epidermal growth factor receptor inhibitor 4. No prior topical or systemic treatment for any other treatment-related or inherent dermatologic disease, including acneiform rash | NR | October 2010-January 2012 | March 2008-October 2009 | Vitamin K1 cream | cetuximab + irinotecan | 1L |
| [Laszlo et al., 2020](https://pubmed.ncbi.nlm.nih.gov/32499108/) | 50,20,30 | Compare the cost and efficacy of granulocyte-colony stimulating factor (G-CSF) plus plerixafor on demand as up-front mobilization regimen to historical control arm of multiple myeloma patients who received cyclophosphamide plus G-CSF as mobilization regimen. | 1. MM patients who were candidates to receive single or tandem autologous stem cell transplantation after a first-line induction and mobilization therapy | NR | May 2015-August 2017 | November 2008- November 2015 | Recombinant human non glycosylated + G-CSF + plerixafor on demand | cyclophosphamide + G-CSF + PBSC | 1L |
| [Lee et al., 2005](https://pubmed.ncbi.nlm.nih.gov/15657178/) | 62,29,33 | Evaluate the effect of alternatively incorporating imatinib into conventional chemotherapy prior to allogenic stem cell transplantation (SCT) for newly diagnosed Philadelphia chromosome-positive acute lymphoblastic leukemia (Ph+ ALL) patients. Additionally, to evaluate the effect of first line (1L) imatinib on allogenic SCT. | 1. Newly diagnosed with Philadelphia chromosome-positive acute lymphoblastic leukemia, completed induction chemotherapy, intended to undergo human leukocyte antigen matched allogenic SCT 2. Karyotypic and/or molecular evidence of t(9;22) in leukemic cells 3. <60 years old 4. Eastern Cooperative Oncology Group performance status of 0 to 2 5. Adequate renal and hepatic functions 6. Adequate cardiac status and negative pregnancy test | 1. Death during induction 2. Lack of donors 3. Old age | September 2000-August 2003 | 1996-2000 | Imatinib interim therapy +induction therapy+ consolidation therapy + salvage therapy | Induction therapy+ consolidation therapy + salvage therapy | 1L |
| [Lienard et al., 1999](https://pubmed.ncbi.nlm.nih.gov/10596916/) | 167,64,103 | Determine whether additional treatment with interferon-gamma (IFNy) is necessary to achieve a complete response rate with isolated limb perfusion (ILP) using tumor necrosis factor-alpha (TNFα) in combination with melphalan in patients with in-transit metastases of melanoma of the limbs. | 1. Histologically confirmed in-transit metastases of malignant melanoma of the limbs (MD Anderson Stage IIIA or stage IIIAB) 2. Progressive disease determined based on tumour measurements 3. Age18-80 years 4. Good performance status (Eastern Cooperative Oncology Group performance status of 0 or 1) | 1. Pregnant or nursing   women   1. Patients with a prior   history of or concurrent malignancy (excluding basal cell carcinoma and in situ carcinoma of the cervix)   1. Patients with systemic   disease or severe lymphoedema of the diseased limb | 1970-1990 | NR | **Intervention #1:** TIM-ILP  **Intervention #2:** TM-ILP | M-ILP | 1L |
| [Martin et al., 2021](https://pubmed.ncbi.nlm.nih.gov/35846215/) | 293,97,196 | Asses the effectiveness of ciltacel from the CARTITUDE-1 trial in comparison to the standard of care using a real-world data of triple-class RRMM | Triple-class exposed  Eastern Cooperative Oncology Group score less than two  Creatinine less than or equal to 2 mg/Dl  Disease progression within 12 months of the most recent lines of therapy (LOTs)  At least three prior LOTs | NR | July 2018-February 2021 | February 2016-February 2021 | Cilta-cel | Physician's choice of treatment (i.e., Carfilzomib, Pomalidomide, Daratumumab, Ixazomib, Elotuzumab, Bortezomib, Lenalidomide, Panobinostat, Selinexor, Thalidomide, or melphalan, or cyclophosphamide c, Dexamethasone alone, among other | ≥4L |
| [Matsuyama et al., 2014](https://pubmed.ncbi.nlm.nih.gov/25502982/) | 94,54,40 | Assess the efficacy and safety of combination immunotherapy with gemcitabine (Gem) and elpamotide in patients with biliary tract cancer. | 1. Pathologically diagnosed   adenocarcinoma or adenosquamous carcinoma with bile duct origin.   1. Unresectable or recurrent disease 2. Aged ≥20 years and <75 years 3. Eastern Cooperative Oncology Group performance status of 0 or 1 -expected to live for ≥3 months 4. Adequate organ function 5. No previous history of chemotherapy, radiotherapy, or immunotherapy for BTC 6. Underwent laparotomy, it was performed ≥2 weeks before registration | 1. Primary disease observably worsened 2. Dose reduction of Gem was required for more than two stages 3. Adverse events made continuation difficult 4. Treatment was postponed for more than 28 days 5. 1.5 years had passed from registration | October 2009-June 2011 | NR | Elpamotide + Gem | Gem without Elpamotide | 1L |
| [Narita et al., 2022](https://pubmed.ncbi.nlm.nih.gov/35649212/) | 75,57,18 | Compare overall survival in treatment-refractory human epidermal growth factor receptor 2 (HER2)-amplified metastatic colorectal cancer (mCRC) patients receiving pertuzumab plus trastuzumab (PER-HER) in the MyPathway study or routine clinical care in the electronic health record-derived real-world data external control arm. | 1. ≥18 years or older 2. Had mCRC with HER2 -Amp status as assessed with comprehensive genomic profiling by Foundation Medicine Inc. treatment-refractory (had received ≥ 2 lines of treatment) 3. Received standard first-line therapy including fluoropyrimidines and either irinotecan or oxaliplatin 4. Received prior treatment other than study drugs or HER2-targeted therapies. | 1. Hematologic malignancies   2. Active or untreated brain metastases  3. History of carcinomatous meningitis 4.Uncontrolled concurrent malignancy  5. Previous treatment with any HER2-targeted therapy  6. Any significant cardiovascular events within 6 months prior to study entry  7. Pulmonary embolism within 30 days prior to study entry, among other criteria | October 2014-Augusst 2017 | January 2011-December 2019 | PER-HER | SOC | Varies (<4L->4L) |
| [O’Haire et al., 2021](https://pubmed.ncbi.nlm.nih.gov/36063280/) | 272,139,133 | Estimate overall survival of advanced and refractory cancer patients participating in the complex genomic profiling (CGP) program compared to synthetic control arm not receiving CGP. | 1.Metastatic or incurable stage patients   1. Eastern Cooperative Oncology Group less than or equal to two 2. Failure of a standard initial line of systemic therapy where this exists 3. Non-synchronous cancers | 1.Colorectal cancer   1. (No CGP cohort only)   Presented to clinic with previous complex testing such as self-funded commercial panels | March 2017- August 2018 | September 2018-September 2019 | CGP | SOC | NA (screening procedure) |
| [Omidvari et al., 2015](https://pubmed.ncbi.nlm.nih.gov/26361613/) | 136,34,102 | Investigate the efficacy of low-dose-rate endorectal brachytherapy (LDRBT) as a boost to neoadjuvant chemoradiation for use in treating locally advanced distal rectal adenocarcinomas. | 1. A newly diagnosed locally advanced (clinical T3–T4 and/or N1/N2, M0) rectal adenocarcinoma 2. No prior therapy 3. Eastern Cooperative Oncology Group performance scale score ≤1 4. Normal or acceptable kidney, liver, cardiovascular, and bone marrow functions 5. Rectal tumors located 10 cm below the anal verge | 1. Rectal tumors at an upper rectal or rectosigmoid location 2. Metastatic disease at presentation 3. Previous history of pelvic irradiation | NR | NR | LDRBT + neoadjuvant chemoradiation | Neoadjuvant chemoradiation without brachytherapy | 1L |
| [Onishi et al., 1996](https://pubmed.ncbi.nlm.nih.gov/9170569/) | 976,37,939 | Compare human leukocyte antigens (HLA) phenotype frequency among advanced renal cell Carcinoma patients who were treated with interferon-alpha. | 1. Diagnosed with renal cell carcinoma 2. Had nephrectomy 3. Had evaluable metastatic lesions | NR | January 1990-April 1994 | January 1990-April 1994 | Interferon-alpha | Placebo | Not reported |
| *[Popat et al., 2022](https://pubmed.ncbi.nlm.nih.gov/35715405/) | 2065,109,1956** | Investigate the comparative effectiveness of pralsetinib against other treatments and demonstrate how we systematically and comprehensively assessed the robustness of the findings against potential biases, serving as a guide for future SCA studies utilizing real-world evidence | 1.1L Pembrolizumab  2. 6 months minimum follow-up  3. No other confirmed mutations  4. No pralsetinib, selpercatinib, or clinical study drug in any other line  5. ECOG=0 or 1  6. non-squamous histology | NR | May 2017- November 2019 | January 2011- September 2019 | Pralsetinib | Scenario 1: 1 L pembrolizumab  Scenario 2: 1 L pembrolizumab with chemotherapy | 1L |
| [Shelikhova et al., 2021](https://pubmed.ncbi.nlm.nih.gov/33836878/) | 257, 149, 108 | Assess the safety and efficacy of low-dose memory (CD45RA-depleted) donor lymphocytes after hematopoietic stem cell transplantation with αβ T-cell depletion | 1. 0-25 years 2. Patients who are considered candidates for allogeneic hematopoietic stem cell transplantation and have one of the following diagnoses: acute lymphocytic leukemia (ALL), acute myeloid leukemia, acute biphenotypic leukemia, acute bilinear leukemia, lymphoma, myelodysplastic syndrome, chronic myeloid leukemia 3. Transplant processing: TCR ab/CD19-depletion | 1. ALL patients not in remission 2. Patients with uncontrolled infections 3. Patients who can perform pulmonary function tests will be excluded if they have a diffusing capacity of the lung for carbon monoxide 4. Unable to perform pulmonary function tests if the oxygen saturation is < 92% on room air, among other criteria | September 2016-August 2019 | January 2014-July 2017 | Tocilizumab | Rabbit antithymocyte globulin | 1L |
| *[Sun et al., 2020](https://pubmed.ncbi.nlm.nih.gov/32535486/) | 1771,272,1499 | The study seeks to contextualize results of the POSITIVE trial assessing the safety of pausing endocrine therapy for young ER+ breast cancer patients desiring pregnancy. It employs two methods based on participants' baseline characteristics, using data from the SOFT/TEXT trials, to estimate standard external breast cancer event rates. | 1.18 and 42 years of age who desired to become pregnant.  2.Completed 18-30 months of adjuvant endocrine therapy for early-stage ER+ breast cancer was eligible to enroll in POSITIVE  3. An interruption of endocrine therapy for up to 2 years is permitted to allow pregnancy attempt (after a 3-month washout period), delivery, and breastfeeding | 1.Had undergone oophorectomy or hysterectomy by 24 months of endocrine therapy | December 2014- December 2019 | 2003-2011 | Interrupting adjuvant endocrine therapy | Adjuvant endocrine therapy | NR |
| [Uffmann et al., 2017](https://pubmed.ncbi.nlm.nih.gov/28400376/) | 237,170,67 | Compare the risk of treatment-related toxicity when lowering the cumulative dose of etoposide to the standard of care in an effort to increase event free survival among patients with Down Syndrome and myeloid leukemia | 1. 4-18 years of age 2. GATA1 mutation detected | Patients with Down syndrome with acute myeloid leukemia French American-British-type M1-M5 | January 2007- August 2015 | August 1998- July 2003 | polychemotherapy + reduced etoposide | polychemotherapy + maintenance therapy | Varies (<4L->4L) |
| [Usmani et al., 2017](https://pubmed.ncbi.nlm.nih.gov/28474745/) | 806,148,658 | Compare the efficacy of daratumumab monotherapy versus historical controls among MM patients. | **Historical Control:**   1. No other cancer diagnosis prior to the diagnosis of MM, except for benign and in situ neoplasms, basal cell carcinoma, and squamous cell carcinoma 2. Received at least 3 prior lines of therapy (LOTs) that included a including a proteasome inhibitor (PI) and an immunomodulatory agent (IMiD) 3. Progression of disease within 60 days of completion of the most recent regimen OR be refractory to both a PI and an IMiD,   **Patients treated with Daratumumab:**   1. ≥18 years of age 2. An Eastern Cooperative Oncology Group performance status ≥2 3. Heavily pretreated and/or refractory MM   **GEN501**: patients were relapsed from or refractory to at least 2 priors, LOTs that included a PI and an IMiD  **SIRIUS**: patients were relapsed from or refractory to at least 3 prior LOTs that included a PI or an IMiD or were double refractory to a PI and an IMiD. | NR | March 2008- May 2014 (GEN501)  September 2013- May 2014 (SIRIUS) | 2000 – 2014 (IMS lifelink)  2007-2014 (OPTUM) | Daratumumab monotherapy | SOC | ≥2L |
| *[Weisel et al., 2021](https://pubmed.ncbi.nlm.nih.gov/34822128/) | 364, 97, 267 | Assess the comparative efficacy of cilta-cel versus physician's choice of treatment in patients with triple-class RRMM | 1. Triple-class exposed  2. Eastern Cooperative Oncology Group score less than two  3. Creatinine less than or equal to 2 mg/Dl  4. Disease progression within 12 months of the most recent lines of therapy (LOTs)  5. At least three prior LOTs | NR | July 2018-February 2021 | June 2014-September 2019 (POLLUX)  September 2014-September 2019 (CASTOR)  February 2014-2019 (EQUULEUS) | Cilta-cel | Physician’s choice of treatment following the discontinuation of study drugs. (i.e., Daratumumab + lenalidomide-dexamethasone and bortezomib-dexamethasone, daratumumab + pomalidomide and dexamethasone, among other) | ≥4L |

i. ALL, acute lymphoblastic leukemia; AML, acute myeloid leukemia; AI, cytarabine, idarubicin; AIE, cytarabine, idarubicin, etoposide; CALND, complete axillary lymph node dissection; CAD, computer-aided detection; CGP, complex genomic profiling; CGP, comprehensive genomic profiling; CT, computed tomography; ESD, endoscopic submucosal dissection; FLT3, fms-like tyrosine kinase receptor-3; FLT3+ AML, acute myeloid leukemia with a FLT3 mutation; G-CSF, granulocyte colony-stimulating factor; Gem, gemcitabine; HA, cytarabine; haM, cytarabine and mitoxantrone; HaE, high-dose cytarabine and etoposide; HER2, human epidermal growth factor receptor 2; HLA, human leukocyte antigens; IFNy, interferon-gamma; ILP, isolated limb perfusion; IMiD, immunomodulatory agent; IMid, immunomodulatory drugs; ITD, internal tandem duplications; LDRBT, low-dose-rate endorectal brachytherapy; LOTs, lines of therapy; LOTs, lines of treatment; mCRC, metastatic colorectal cancer; MX, metastasis cannot be measured; NR, not reported; NX, cancer in nearby lymph nodes cannot be measured; OS, overall survival; PBSC, peripheral blood stem cell; PER-HER, pertuzumab plus trastuzumab; PI, proteasome inhibitor; RRMM, relapsed or refractory multiple myeloma; SCT, stem cell transplantation; SLN, sentinel lymph node; TKD, tyrosine kinase domain.

* Article manually added

**Sample size from [Gökbuget et al.](https://www.ncbi.nlm.nih.gov/pmc/articles/PMC5056974/pdf/bcj201684a.pdf) n=1139 is divided into 1112 for the overall survival analysis, and 694 for the complete remission analysis; sample size from [Popat et al.](https://pubmed.ncbi.nlm.nih.gov/35715405/) n=1956 is divided into 686 for Pembrolizumab, and 1270 for Pembrolizumab + chemotherapy
